# Supplementary material for: Analysis of epidemic trend of respiratory pathogens in children after long-term pathogen isolation
Source: PeerJ. 2025 Jul 16;13:e19710. doi: 10.7717/peerj.19710 (PMC12275896; doi:10.7717/peerj.19710)
Supplement: Supplemental Information 3 [file peerj-13-19710-s003.docx]

MIQE specification file

This study was for clinical specimen detection and result analysis, and no control group was involved. The experimental data involved 9670 specimens.

The specimens in this study were sputum samples or throat swabs from clinical patients.

This study did not involve any pre-treatment steps related to case specimens

The reagents used in this study are commercial reagents from Shengxiang Biotechnology Co., LTD.

1. Extraction reagent: nucleic acid extraction or purification reagent (S10015),It is used for nucleic acid extraction, enrichment, purification and other steps, and the processed products are used for clinical in vitro detection. After the separation of the sample containing the target nucleoacid, the magnetic beads can be identified and efficiently combined with the DNA/RNA molecules, and the magnetic beads are adsorbed on the tube wall by the magnetic separator, and the high purity is obtained through the washing, elution and purification process.

DNA/RNA.

Extraction reagent composition

| Serial number | Reagent name | Specification |
| --- | --- | --- |
| 1 | Extraction solution | 500μL/hole/piece |
| 2 | Washing solution 1 | 750μL/hole/piece |
| 3 | Washing solution 2 | 750μL/hole/piece |
| 4 | eluate | 80μL/hole/piece |

1. Amplification reagents: ① six respiratory tract pathogenic bacteria nucleic acid detection kit (multiple fluorescent PCR method), this kit qualitative detection of human sputum clinical common lower respiratory tract pathogens, including Klebsiella pneumoniae, streptococcus pneumoniae, Haemophilus influenza capsulatus, pseudomonas aeruginosa, Legionella pneumophila and staphylococcus aureus in vitro qualitative detection. This kit is based on the two technical principles of Taq enzyme hydrolysis fluorescence probe to produce fluorescence signal and hybridization of fluorescent products to produce fluorescence signal. For the detection of one target in the monochromatic fluorescence channel, fluorescence probe is used, and the detection of the other target is by the way of melting curve. Thus, the detection and analysis of two targets in the monochromatic fluorescence channel can be realized simultaneously. Specific primers and specific fluorescent probes designed for the conserved area of bacterial nucleic acid to be detected are combined with PCR reaction solution and other components. Multiple real-time fluorescent quantitative PCR detection technology is applied on the fluorescence quantitative PCR instrument to achieve rapid detection of pathogenic bacterial nucleic acid in the lower respiratory tract of tested samples through changes in fluorescence signals.

The PCR detection system contains a positive internal control (internal standard). By detecting whether the DNA encoding the butler gene of glyceraldehyde-3-phosphate dehydrogenase (GAPDH) in the sample to be tested is normal, the extraction process and PCR amplification process of the sample to be tested can be monitored to avoid false negative results.

| Serial number | Constituent | Capacity | Main component |
| --- | --- | --- | --- |
| 1 | PCR reaction solution | 1056μL/tube | Primers, probes, dNTPs, PCR buffers, etc |
| 2 | Enzyme mixture | 24μL/tube | Taq enzyme,UDG enzyme |
| 3 | Positive control | 1000μL/tube | Plasmid |
| 4 | Negative control | 1000μL/tube | Normal saline |

Analysis of results:

Cycle parameter setting:

| Procedure | | Temperature | Time | Cycle number |
| --- | --- | --- | --- | --- |
| 1 | UDG enzyme reaction | 50℃ | 2min | 1 |
| 2 | Predegeneration | 94℃ | 3min | 1 |
| 3 | Degeneration | 94℃ | 10s |  |
| 4 | Anneal | 60℃ | 20s | 45 |
| 4 | Elongation and fluorescence detection | 75℃ | 20s |  |
| 5 | Solution curve | 62-75℃ | Whole-process acquisition fluorescence | 1 |

(1) The detection signal of the target is FAM, HEX (or VIC), ROX, and CY5 channels, and the detection signal of the internal standard is the melting curve of the HEX channel;

(2) Baseline setting: Baseline is generally set to 3-15 cycles, which can be adjusted according to the actual situation. The adjustment principle is as follows: Select the region where the fluorescence signal is more stable before exponential amplification, the starting point avoids the signal fluctuation in the initial stage of fluorescence acquisition, and the end point is 1-2 cycles less than that of the sample Ct with the earliest exponential amplification. Setting of Threshold: The setting principle is that the threshold line is just above the highest point of the normal negative control product.

(3) Determination of negative results: First analyze whether the internal standard detects the characteristic peak of the melting curve in the HEX channel, and the Tm is between 65.8 and 68.5℃. If yes, it indicates that the detection is effective and subsequent analysis can be continued:

i. If the typical S-type amplification curve was detected in the FAM channel and Ct≤39, the detection result of Klebsiella pneumoniae was positive; if Ct > 39 or no Ct, it was negative; If the characteristic peak of Tm (68.8~71.3℃) was detected in FAM channel, the detection result of Legionella pneumophila was positive; if there was no characteristic peak of melting curve or Tm was not between 68.8~71.3℃, it was negative.

ii. If HEX channel detects a typical S-shaped amplification curve and Ct≤39, Streptococcus pneumoniae test result is positive; if Ct > 39 or no Ct, it is negative;

iii. If the typical S-type amplification curve is detected by the ROX channel and Ct≤39, the detection result of Haemophilus influenzae is positive; if Ct > 39 or no Ct, it is negative;

iv. If the typical S-type amplification curve is detected in CY5 channel and Ct≤39, the detection result of Pseudomonas aeruginosa is positive; if Ct > 39 or no Ct, it is negative; If the characteristic peak of Tm (66.8~70.0℃) is detected by CY5 channel, it means that the detection result of Staphylococcus aureus is positive; if there is no characteristic peak of melting curve or Tm is not between 66.8~70.0℃, it is negative;

v. If the internal standard does not detect the characteristic peak of Tm (65.8~68.5℃) in the HEX channel, it means that the concentration of the tested sample is too low or there is interference substance inhibition reaction, and the experiment needs to be prepared again.

Product performance index:

Positive and negative conformity rate: This kit detects enterprise reference products, positive and negative conformity rate is 100%.

Minimum detection limit: The minimum detection limits for each pathogen enterprise reference products of this kit were Klebsiella pneumoniae 900 CFU/mL, Streptococcus pneumoniae 15 CFU/mL, Haemophilus influenae 625 CFU/mL, Pseudomonas aeruginosa 675 CFU/mL, Legionella pneumophila 340 CFU/mL and Staphylococcus aureus respectively 2875 CFU/mL.

Precision: The precision test showed that the detection repeatability was good between batches, during the day, between different operators and in different rooms. The coefficient of variation (CV, %) of Ct value of parallel detection results of the same sample should be less than 5%, and the detection results of the melting curve should be consistent, all of which were positive.

Specificity: There is no cross-reaction among respiratory tract pathogens covered by this kit; With common respiratory pathogens (Staphylococcus haemolyticus, Staphylococcus epidermidis, Acinetobacter baumannii, Escherichia coli, Serratia marcescens, stenotrophomonas maltophilia, Enterococcus faecalis, Candida albicans, Klebsiella acidogenes, Streptococcus pyogenes, Micrococcus flavus, Rhodococcus equi, Listeria grii, Acinetobacter Joni, Haemophilus parainfluenza, Legionella Dumov, aerogenic intestine) No cross-reactivity was observed in positive samples of Bacillus, Haemophilus haemolyticus, oligopurticomonas maltophilum, Mycoplasma pneumoniae, Chlamydia pneumoniae, Streptococcus salivarius, Neisseria meningitidis, Mycobacterium tuberculosis, influenza A virus, influenza B virus, Aspergillus flavus, Aspergillus aspergillus, Candida glabrata, Candida tropicalis).

Anti-interference ability: The detection of reference substances for interfering substances showed that common drugs for the treatment of respiratory tract infections (such as meropenem, imipenem, cefoperazone sulbactam, moxifloxacin, amikacin, linezolid, vancomycin, etc.) did not interfere with this reagent at normal dose concentrations.

② Six nucleic acid detection kits for respiratory pathogens (PCR-fluorescent probe method). This kit is used for qualitative detection of nucleic acids of influenza A virus, influenza B virus, respiratory syncytial virus, adenovirus, human rhinovirus and mycoplasma pneumoniae in human throat swab samples. This kit uses specific primers and specific fluorescent probes designed for the conserved region of nucleic acid of pathogens to be detected, coupled with PCR reaction solution and other components. Multiple real-time fluorescent quantitative PCR detection technology is applied on the fluorescence quantitative PCR instrument to realize rapid detection of nucleic acid of respiratory pathogens in samples through changes in fluorescence signals. The PCR detection system contains a positive internal control (internal standard). The extraction process of the sample to be tested and the PCR amplification process are monitored by detecting whether the human butler gene encoding glyceraldehyde-3-phosphate dehydrogenase (GAPDH) in the sample is normally amplified to avoid false negative results.

| Serial number | Constituent | Capacity | Main component |
| --- | --- | --- | --- |
| 1 | PCR reaction solution A | 1044μL/tube | Primers, probes, dNTPs, PCR buffers, etc |
| 2 | PCR reaction solution B | 1044μL/tube | Primers, probes, dNTPs, PCR buffers, etc |
| 3 | Enzyme mixture | 24μL/tube | Taq enzyme,UDG enzyme |
| 4 | Positive control | 1000μL/tube | Plasmid |
| 5 | Negative control | 1000μL/tube | Normal saline |

Analysis of results:

1. PCR mixture A
2. 1) FAM channel (Reporter: FAM, Quencher: none) was selected to detect influenza A virus nucleic acid. 2) HEX or VIC channel (Reporter: HEX/VIC, Quencher: none) was selected to detect influenza B virus nucleic acid; 3) CY5 channel (Reporter: CY5, Quencher: none) was selected to detect RSV nucleic acid; 4) Select ROX channel (Reporter: ROX, Quencher: none) to detect internal standard nucleic acid. Set the Sample Volume to 50.
3. 2) PCR mixture B
4. The FAM channel (Reporter: FAM, Quencher: none) was selected to detect adenovirus nucleic acid. 2) Select HEX or VIC channel (Reporter: HEX/VIC, Quencher: none) to detect human rhinovirus nucleic acid; 3) CY5 channel (Reporter: CY5, Quencher: none) was selected to detect mycoplasma pneumoniae nucleic acid. 4) Select ROX channel (Reporter: ROX, Quencher: none) to detect internal standard nucleic acid. Set the Sample Volume to 50.

Cycle parameter setting:

| Procedure | | Temperature | Time | Cycle number |
| --- | --- | --- | --- | --- |
| 1 | Reverse transcription | 50℃ | 30min | 1 |
| 2 | Predegeneration | 95℃ | 1min | 1 |
| 3 | Degeneration | 95℃ | 15s | 45 |
| 4 | Annealing, elongation and fluorescence detection | 60℃ | 30s |  |
| 5 | Instrument cooling | 25℃ | 10s | 1 |

After the reaction is over, the results are automatically saved, and the Baseline Start value, End value and Threshold value are adjusted according to the image analysis (users can adjust the baseline according to the actual situation, the Start value can be set at 3 ~ 15, and the End value can be set at 5 ~ 20). Adjust the amplification curve of the negative control to be flat or lower than the threshold line), click Analyze for analysis, and record the Ct value.

| Amplification result | FAM passage | | HEX passage | | CY5 passage | | ROX passage | |
| --- | --- | --- | --- | --- | --- | --- | --- | --- |
|  | Ct≤40 | No Ct or Ct＞40 | Ct≤40 | No Ct or Ct＞40 | Ct≤40 | No Ct or Ct＞40 | Ct≤40 | No Ct or Ct＞40 |
| PCR reaction solution A | Flu A positive | Flu A negative | Flu B positive | Flu Bnegative | RSV positive | RSV negative | Internal label positive | Internal label negative |
| PCR reaction solution B | HADV positive | HADV negative | HRV positive | HRV negative | MP positive | MP negative |  |  |

Performance index:

This kit for the detection of influenza A/B virus nucleic acid detection reagents national reference: positive coincidence rate (+/+) is 6/6; The negative coincidence rate (-/-) was 6/6; Precision (CV, %) : CV is less than 5.0%. Minimum pathogen detection limit: Influenza A virus 2.0TCID50/mL, adenovirus 500.0copies/mL, influenza B virus 2.0TCID50/mL, Mycoplasma pneumoniae 500.0copies/mL, respiratory syncytial virus 500.0copies/mL, human rhinovirus 500.0copies/mL.

3. Experimental instruments

① Extraction instrument: NATCH 48 nucleic acid extraction instrument of Shengxiang Biotechnology Co., LTD

② Nucleic acid amplification instrument: SLAN-96P type nucleic acid amplification instrument of Hongshi Biotechnology Co., LTD

4. Experimental steps

This study did not involve nucleic acid concentration determination and electrophoresis

① Sample pretreatment

Add equal volume normal saline or 4%NaOH into the sample collection tube, fully liquify, and then pour all the liquid into the 1.5mL centrifuge tube as the sample to be measured.

② adding sample

Take the prepared samples of 300μL and add them into the extraction liquid plate successively.

③ Machine extraction

Place the extraction liquid plate, washing liquid 1 plate, washing liquid 2 plate and eluent plate with the sample added into the extraction instrument in turn, open the extraction procedure and start extraction.

The procedure is set as follows: the extraction plate is shaken and mixed for 30S, and heated at 60℃ for 10min. Stand still for 1min, magnetic separation, transfer the sample to washing liquid 1 plate, shake and mix for 30s, and then place it in the magnetic separator again. After magnetic absorption for 3min, transfer the sample to the washing liquid plate 2, shake and mix for 30s, and then place it in the magnetic separator again. After magnetic absorption for 3min, transfer the sample to the eluent, shake and mix for 30 30S, and let stand for 3min.

④ Reaction liquid configuration

The amplification reagent was balanced to room temperature, and the reaction liquid was 43.5μL/ person + 1.5μL/ person mixture of H6 enzyme, and the reaction system was configured.

⑤ The extracted sample of 5μL was added to the reaction system, and then centrifuged after shock, the machine was used to calculate the amplification instrument.

5、Primer information

| Target virus | Gene | Final concentration | Amplification size(bp) |
| --- | --- | --- | --- |
| IFV-A | M gene | 1 uM | 270 |
| IFV-B | M gene | 1 uM | 166 |
| RSV | Large Polymerase L gene | 1 uM | 158 |
| HAdV | Hexon gene | 1.75 uM | 338 |
| HRV | 59-UTR | 1 uM | 144 |
| MP | Cytadhesin proteir p1 gene | 1.5 uM | 156 |
| SA | Nuc gene | 1.75 uM | 182 |
| KP | SHV gene | 2uM | 208 |
| LP | Mip gene | 1 uM | 229 |
| HI | bexA gene | 1.5 uM | 278 |
| PA | Oprl gene | 2 uM | 219 |
| SP | ply gene | 1 uM | 174 |

The statistical software is SPSS Statistics 23
